# Climate and development: enhancing impact through stronger linkages in the implementation of the Paris Agreement and the Sustainable Development Goals (SDGs)

**DOI:** 10.1098/rsta.2016.0444

**Published:** 2018-04-02

**Authors:** Luis Gomez-Echeverri

**Affiliations:** Transitions to New Technologies, International Institute for Applied Systems Analysis, Schlossplatz 1, 2361 Laxenburg, Austria

**Keywords:** Sustainable Development Goals, Paris Agreement, governance, political economy, climate and development linkages, decarbonization

## Abstract

One of the greatest achievements in the global negotiations of 2015 that delivered the 2030 Agenda for Sustainable Development or Sustainable Development Goals (SDGs) and the Paris Agreement on climate change is that, for the first time, the linkages between climate and development were enshrined in each of the documents. This was done in recognition that climate change and development need to be addressed together in order not only to avoid harmful trade-offs and high costs, particularly for poorer countries, but also to exploit the benefits that come from strengthening these linkages. This review presents some of the latest data that argue for stronger linkages as well as the challenges of implementation which are not only politically and economically related but also include issues such as knowledge gaps, finance and governance. Finally, the review also presents a glimpse at the pathways that will be required to reach the ambitious global temperature targets of the Paris Agreement of less than 2°C above pre-industrial levels with efforts to limit temperature rise even further to 1.5°C. This provides the context for some conclusions and recommendations for policy-makers, including on methodologies for assessing linkages and leveraging them for greater benefit.

This article is part of the theme issue ‘The Paris Agreement: understanding the physical and social challenges for a warming world of 1.5°C above pre-industrial levels’.

## 1. Introduction

Two major achievements in global negotiations are likely to have a significant transformative effect on the global development agenda for decades to come. The Sustainable Development Goals (SDGs) [1] and the United Nations Framework Convention on Climate Change (UNFCCC) Paris Agreement [2] aspire to transform the way in which development issues and climate change are addressed. For the first time ever, there is an agreement by the international community on priority areas for development with an actionable agenda and a global commitment to reach certain targets and goals between now and 2030. The 17 SDGs constitute an agenda that seeks to balance the three dimensions of sustainable development—social, economic and environmental—as well as issues of governance and institutions [3]. This is in recognition of the fact that it is difficult, if not impossible, to achieve socio-economic gains if the environment, and threats such as climate change, are neglected; and that promoting synergies and mitigating or eliminating trade-offs among SDGs is essential for achieving this balance and for helping achieve the temperature targets of the Paris Agreement. In this regard, understanding how the various SDGs are interlinked is crucial to the formulation of good policies and more effective action and finance in support of implementation.

One of the messages of this review is that the success of each of these two global agreements will depend heavily on the other and on the capacity of countries to develop and implement programmes of action to address their climate and development objectives in an integrated, coordinated and comprehensive way. Institutions, policies, governance and finance will need to play key roles in support of these efforts. Through their 17 global goals and 169 targets, the SDGs provide the most comprehensive and balanced global development agenda to date to help countries meet the immense current development challenges without compromising their chances to adequately address climate change [4]. The objective is to be able to meet the growing needs of development while making sure that low-carbon transition goals are incorporated into the development agenda [5]. Asia and Africa particularly face some of the biggest challenges. Africa, where greenhouse gas (GHG) emissions *per capita* are the lowest because of historical poverty trends and stagnation, is now growing and some of its cities count among the fastest growing in the world [6]. This will bring higher GHG emissions unless policies and strategies are put into place to mitigate them. It is an excellent opportunity for the continent to embark on a low-carbon development path using its immense resource endowments suitable for renewable energy. Asia, the continent that currently emits 40% of the global GHG emissions, which are due to continue rising by some 50% if policies are not put into place, has the highest growth in urban population; this is expected to be around 67% by 2030. Major investments will be needed to meet the demand of this growing population. The Asian Development Bank projects that the incremental cost of introducing low-carbon development compatible with the Paris Agreement will be of the order of some US$300 billion per year through 2050 [7].

One of the SDGs, SDG 13, specifically addresses climate and it calls on the international community to ‘take action to combat climate change and its impacts’. Its targets address issues of implementation such as planning, policies, institutional and individual capacity building, and, most importantly, issues of finance. The areas that it addresses for action—mitigation, adaptation and resilience—are linked to several SDGs that either impact on climate change (such as energy, industry and infrastructure, production and consumption, and sustainable cities) or are impacted by climate change (such as poverty, food security, and health among others). As for the Paris Agreement, for the first time ever, it includes a global commitment for domestic action through the nationally determined contributions (NDCs), coupled with a promise and commitment to mobilize climate finance to support those countries which need it. The NDCs represent a major turning point in the fight against climate change. The process of preparing them in the lead up to the UNFCCC Paris Conference of the Parties (COP21) has already triggered a major mobilization of national consultations, debate and interest, and expectations have been raised. In some countries, special institutional arrangements for implementation of the NDCs have begun to emerge. The questions that need to be explored are whether these emerging arrangements and/or old existing ones will be up to the task of ensuring an effective implementation and whether the commitments are sufficiently ambitious.

According to the most recent United Nations Environment Programme (UNEP) Emissions Gap Report [7], current commitments by countries through their NDCs fall short of what is required to meet the ambitious goals of the Paris Agreement. The data in this UNEP report show that the world is on its way to a temperature rise of 2.9–3.4°C even when accounting for the current NDC pledges. A study conducted by the Asian Development Bank [8] estimates that the commitments made in the NDC pledges by Asia, which is the fastest growing region, are far from being sufficient for the Paris Agreement requirements. Current NDCs commit to halve the GHG emissions by 2050. The Asian Development Bank estimates that, to meet the targets, the reductions would need to be around three-quarters. The process established to periodically review and strengthen these pledges will need therefore to address these deficits. This is a pre-requisite for the success of the Paris Agreement. The first review will take place in 2018 and every 5 years thereafter. Each of these will be an opportunity to ramp up the ambition as well as the political will and support needed based on assessments made.

For these UNFCCC periodic reviews to be successful, research will also need to be ramped up so that policy-makers have clarity not only on the implications of temperature rise, costs of inaction and the windows of opportunity but also on the options for action. Thus, the Intergovernmental Panel on Climate Change (IPCC) special assessment report on the science of 1.5°C to be published in 2018 will be very timely. Given the ambition of the goals, all stakeholders will need to contribute. The research and science will need to provide answers that are relevant to different constituencies. One crucial constituency includes sub-national and city leaders. Data and research need to be offered so that they can assess the implications of climate change and the types of action that can be taken in the short, medium and long term.

## 2. Climate and development linkages

Over the past two decades, a large body of literature has offered important insights into the socio-economic costs and benefits of addressing climate change. The results provide strong justification for the urgent need to carefully examine the linkages between climate and development to exploit the synergies and avoid or mitigate the negative trade-offs. The intention here is not to create a comprehensive list of the literature but rather to present a selection to reinforce the argument that the success of the Paris Agreement is dependent on understanding and acting on these linkages.

The following is a sample of the literature and some of the messages and conclusions presented. These include the global assessments of the science and the literature on the global and sectoral impacts of climate change; reports that assess the costs of climate change to the global economy, a key piece in the puzzle of how climate change will actually shape the global economy so that policy-makers can gauge the level of investments in emissions reduction to counteract the negative effects; the cost of inaction, which has helped to emphasize the urgency and the magnitude of the investments required to avoid higher costs, and the windows of opportunity for taking these measures in the most cost-effective manner; the co-benefits that come from taking these measures, particularly for ecosystems and human health, which have been important incentives for policy-makers to invest in climate change measures particularly at the national level; assessments of impacts at the regional level, mostly on agriculture and the hydrological cycle; national studies with more tailored research into the impacts of climate change on ecosystems, economies and societies such as those of Austria, Australia and Uganda mentioned below; studies on the effects on the value of investments and financial assets to help investors carry out relevant risk assessment and studies on how climate change has already impacted and could impact the levels of migration in the future, as well as peace and conflict.

The recent 5th Assessment Report of the IPCC provides one of the most comprehensive global and sectoral assessments of the impacts of climate change on development, the levels of impact and the degrees of confidence [9]. In its assessment, the IPCC provides a broad view of impacts on key sectors for development and well-being, including, among others, food security, urban systems and the effects on key economic sectors such as energy, water, transport, human health, human security and poverty. The most important conclusion is that climate-related hazards aggravate factors that bring negative consequences, particularly to people living in poverty. The result, the IPCC concludes, is that climate change makes existing poverty worse, leading to more inequality and more vulnerability. It also concludes that climate change ‘will create new poor between now and 2100, in developing and developed countries, and jeopardize sustainable development’ [9].

On the global economy, a study recently published in *Nature* shows that the impact on the global economy due to rising temperatures is greater than previously assessed. According to this study, the effects include a significant fall in incomes in most countries by the year 2100 under a business-as-usual scenario [10]. Another recent study published by the Organisation for Economic Co-operation and Development (OECD) [11] assesses the consequences of climate change for a selected number of drivers of growth, including productivity, capital supply and labour. The quantitative assessments made to the year 2060 conclude that the greatest negative effects projected are for the agricultural and health sectors, and the greatest damage to take place will be in developing countries, particularly Africa and Asia. Less recent but interesting studies look at the potential negative consequences of climate policy for the economy and how to offset them with a focus on employment effects [12,13]. In these studies, the authors examine the political economy aspects of emissions reduction policies and the short-term considerations that often dominate these discussions. A more balanced approach, they argue, would take the longer view and include policies designed to mitigate or avoid the negative effects that could result from restrictive emissions reduction policies.

One stream of research has been focusing on the costs resulting from a lack of action or delayed action and the severe effects on poorer countries. In this category, one of the best known reviews that helped to shape and influence thinking and research over the decade leading to the Paris Agreement is the Stern Review [14], which concluded, among other things, that all countries would be affected but that developing countries would be the hardest hit and sooner than most. The Stern Review predicted that this would happen when different events interacted to compound the effects on welfare: disruptions to the water cycle, health aspects, reductions in agricultural production and an increase in the number of weather events, making adaptation more difficult and costly [15]. The Review pointed out the potential major and lasting damage to human life, health and the environment, but also the effects on productive capital and consequently on economic growth. On a more positive note, the Review pointed out that disastrous climate change impacts are still avoidable if we act now rather than later, when the costs will be higher, and with increased international cooperation. The Review also focused on the costs of inaction and, most importantly, argued that these costs would be greater than the costs of action. Unabated climate change, the Review showed, would lead to a cost to the world economy of some 5% of gross domestic product (GDP). However, the cost of reducing emissions could be limited to some 1% of GDP. In a recent lecture to celebrate 10 years since the publication of the Review [16], the lead author updated some of the main messages and conclusions. The following are some of the highlights of these updates: science has advanced and shown that the risks are even greater than previously thought; GHG emissions per year have grown instead of decreased; there is greater recognition of the economics of climate change and low-carbon growth opportunities; the concept of ‘cost of action’ has been transformed by the major technological breakthroughs, particularly in renewable energy and energy efficiency, bringing a welcome shift from the notion of ‘costs’ to one of ‘investment’ that has created a world of new opportunities; political progress is slowly picking up particularly since the Paris Agreement; bottom-up action has increased with more active sub-national actors and private sector engagement; and, finally, there is welcome news that there is growing recognition that sustainable development, poverty reduction and climate are inextricably linked.

One study by the Economist Intelligence Unit [17] focused on the impact of climate change on current managed assets and found that the average value at risk of these assets from climate change is some 3% of the current value. A study carried out later by the Grantham Research Institute on Climate Change and the Environment updated some of these estimates using an integrated assessment model to assess the possible impacts on the current market value of global financial assets up to 2100 and came up with somewhat equivalent, albeit slightly lower, results [18]. Two studies, one by International Institute for Applied Systems Analysis (IIASA) researchers, modelled up to 2050, and one other published in *Nature Climate Change*, studied the effects of reducing emissions on co-emitted air pollutants, which in turn brings benefits to health [19,20]. The IIASA study shows how stringent climate mitigation strategies have co-benefits for air quality and the resulting positive effects linked to human health and reduction of loss of life expectancy in Europe, India and China by 35%, 46% and 63% respectively; and for ecosystems in Europe with the lowering of the impact of acidification exposure on forests and less eutrophication due to less ammonia emissions from agriculture. The study published in *Nature Climate Change* shows the significant benefits of improved air quality and the resulting positive impacts on health and air pollution mortality resulting from GHG emissions reductions. One of the main messages is that, since these effects and impacts are mostly local and near-term, they may be useful to policy-makers trying to promote GHG emissions reductions and stringent climate strategies.

Regional and national studies have offered important assessments of the impacts of climate change not only on ecosystems but also on the economy and society. Some of these include the study produced by the Austrian Panel on Climate Change [21], which consists of a group of some 200 scientists, over a 3 year period followed the methodology of the IPCC and has enabled policy-makers to strengthen Austria's strategy on climate change; the Garnaut Review [22], Australia's comprehensive review of the impacts of climate change for Australia and an assessment of the contributions that Australia could make globally; the economic assessments made on the impact of climate change on Uganda [23], which estimated that damage to agriculture, infrastructure, energy and water could total some 2–4% of GDP during the period 2010–2050 and that adaptation costs were high but the costs of inaction significantly higher by some 20–40 times; studies that have provided important methodological advances and analysis based on scenarios for the twenty-first century on the impacts of climate change on the hydrological cycle, such as the one that estimated the effects on water management in India [24] and the impact of climate change on water resource quantity and quality indicators for a region in Greece [25]; studies that have estimated the impacts on climate from the bio-geophysical (BGP) and bio-geochemical (BGC) effects of land use change in different regions of the world where close to one-third of the global land surface has already suffered from deforestation and introduction of cropland and pastures [26]; and studies on ocean acidification and its effect on carbon uptake in different regions of the world due to decreased carbon dioxide solubility and local warming due to large freshwater fluxes, among others [27].

The impacts of climate change on the agricultural sector affecting food security have been more problematic. The general conclusion is that climate change has reduced crop yields by some 1–2% in each decade over the last century. The projected impacts are expected to worsen in the future [9]. It has been difficult to have a good understanding of the nature and magnitude of the impacts because of the number of socio-economic and biophysical factors involved. Recent literature has made advances using shared socio-economic pathways (SSPs), and recent studies have concluded that the biophysical agricultural productivity will be negatively impacted by climate change in most parts of the world. This therefore justifies mitigation efforts. The studies also concluded that regions will be affected differently, with some crop yields being affected negatively and others positively depending on the location [28,29].

A scarcer but rapidly growing body of literature is that which includes studies on migration and peace and conflict. On migration, studies range from those such as a recent article in *Nature*, which makes some inferences on early human migration based on computer modelling of climate effects [30], to more localized studies such as that carried for Mexico during the decade 1995 to 2005 during which climatic conditions led to severe crop failures and greater than normal emigration across the border to the USA [31]. The conclusions of this study could be quite applicable to today and to other regions of the world. Although the total number of people displaced because of environmental change is not well known, there are some estimates. A study by the International Organization for Migration (IOM) [32] estimates that, in 2008, there were more people displaced by extreme weather events (some 20 million) than by conflict and violence (about 4.6 million). On peace and conflict, one of the most informative studies is that which appeared recently in *Science* [33] and which examined a vast array of literature on many types of human conflict. Focusing on quantitative studies that could reliably show causal associations between climate variability and conflict, the authors show that there is significant agreement among the studies on the influence of climate on human conflict across temporal and spatial scales and across several regions of the world.

There is also a more recent growing body of literature examining interlinkages and methodologies for measuring these interlinkages between SDGs, including SDG 13 and those of most other SDGs [3,4,34–36]. In its Review of Targets for the Sustainable Goals: The Science Perspective, the International Council for Science (ICSU) points out that the lack of proper response to climate change would most likely make it more difficult to achieve targets related to many SDGs such zero hunger, good health and well-being, clean water and sanitation, economic growth, industry, innovation and infrastructure, sustainable cities, and life on land and below water. Poverty, inequality and peace and justice would also be indirectly affected [3]. In a more recent publication, the ICSU presents the work by a group of scientists who analysed interactions and synergies of four SDGs—SDG 2 on Zero Hunger, SDG 3 on Quality Education, SDG 7 on Affordable and Clean Energy, and SDG 14 on Life Below Water. Although this was not focused on climate and development interactions, the study did show synergies with SDG 13 on Climate Action and others. The study offered a methodology for assessing the interactions based on a seven-point scale to measure positive interactions as well as interactions that could bring trade-offs [37].

The World in 2050 [38], a new major research initiative in support of the implementation of the SDGs and the Paris Agreement, presents the following in its vision statement: ‘There is an urgent need for a truly integrated, comprehensive quantitative understanding of sustainable development pathways, accounting for the interlinkages between the economy, technology, environment, climate, human development and planetary boundaries.’ And, in its concept-note, it goes on to add, ‘The currently used long-term projections for the world economy do not tend to account for the impact of climate change or different demographic developments. Similarly, models for climate change mitigation are poorly integrated with models for biodiversity as well as the use of land and water resources. Moreover, we lack a proper understanding of the interrelations between policies aimed at productivity growth, material welfare, energy access, and environmental sustainability’ [38]. There are, therefore, ongoing efforts by this initiative to integrate some of the world-class models in key sectors such as those for global food policy (IMPACT/GLOBIOM/GAEZ), ecosystem changes (IMAGE), climate and energy (MESSAGE) and more recent modelling advances in Earth System Science and economic modelling.

## 3. Pathways to implementation—some of the main drivers, what action needs to be taken and on what time scales

The long-term temperature target of the Paris Agreement is ambitious but, according to the best available science, feasible. It is, however, extremely challenging given that it requires emissions to peak as early as possible and to reach zero some time around the middle of the century [39]. To succeed with a 1.5° pathway, a major transformation of systems, particularly energy systems, is required across sectors and geographies as well as major collaboration globally. A publication by *Nature Climate Change* [40] presents some of the main key elements of the energy system transformation and when they need to take place. Decarbonization of the electricity system is listed as a top priority. In this context, electricity would need to emit close to zero carbon emissions by 2050 at the latest. The industry, buildings and transport sectors are singled out as those that need greater efforts to decarbonize, with one of these sectors, transport, being particularly difficult due to its dependence on liquid fossil fuels. Another important element in the transformation of the energy system is the urgent need to reduce energy demand through greater efficiency. This therefore includes the need for dedicated energy efficiency policies to accelerate energy intensity reductions. For those sectors that are harder to decarbonize, such as transport and industry, carbon dioxide removal (CDR) technologies are seen as essential for 1.5°C scenarios to compensate for residual emissions from these sectors.

Scenarios have been important tools of research on climate and global environmental change. There is ongoing effort to develop a new family of scenarios that seek to integrate changes in climate as well as changes in society in order to determine not only the impacts of climate but also the various available options for mitigation and adaptation [41,42]. Given the close linkages between climate and development, the design of these new tools of research is essential and urgent. One of the features of this new family of scenarios is the so-called shared socio-economic pathways (SSPs). By describing a set of alternative futures, and back casting from a set of desired narratives, these SSPs provide descriptions of plausible future conditions with different combinations of challenges to mitigation and adaptation. As work on these SSPs continues and the tools and framework of analysis become more refined, pathways to implementation that incorporate not only quantitative components but also qualitative narratives for those issues that are difficult to quantify will hopefully become more common.

The refinement of these tools is essential to help researchers come up with priorities for action, particularly those involving the big drivers and megatrends that will determine future sustainability with major socio-economic consequences. These include, among others, the growth of energy demand, the food security imperative, the water resilience challenge, and the growth of urbanization. Of these, the central and most important for the Paris Agreement is the need for transformation of the energy systems, as already mentioned. The encouraging news in this regard is the continued improvement in the energy intensity of the global economy and the growth in the deployment of renewable energy worldwide as well as the projected continued growth [43,44]. In its most recent World Energy Outlook, the International Energy Agency (IEA) sums up the trends in deployment and costs of clean energy technologies as follows: ‘in 2016, growth in solar PV [photovoltaic] capacity was larger than for any other form of generation; since 2010, costs of new solar PV have come down by 70%, wind by 25% and battery costs by 40%’ [44].

Is this encouraging news and developments sufficient? According to projections by the IEA, some US$44 trillion in energy supply infrastructure will be required based on its main scenario (to 2040). Of these, the IEA projects that some 20% will be renewable energy. Although this represents a major shift in capital investments, the study also shows that this is not enough for the less than 2°C target of the Paris Agreement. In its more stringent scenario required by a more decarbonized energy system, the IEA projects additional investment in renewable energy and added investments in energy efficiency. The report also points out another important trend in the nexus between water and energy, A rising population will increase the demand for water in urban populations, which in turn will cause a rise in energy demand. The energy use demand in the water sector is expected to double during the period up to 2040 [45]. In these and other energy demand analyses, the impact of infrastructure in cities is most prominent. Consequently, the conclusion is that the Paris Agreement's target cannot be reached without a major transformation of energy systems in cities, particularly in its buildings and water, energy and transport infrastructure.

Cities are projected to add some 2.5 billion residents by 2050 [46], and currently they are responsible for more than 70% of carbon dioxide emissions [46]. Cities, therefore, need to be a central part of the effort to reach the Paris Agreement targets as they offer the best opportunities for decarbonization. And cities in developing countries would need to be a major focus as 90% of the urban growth to 2050 is expected to take place mostly in Asia and Africa, where there is less capacity to address climate change. The challenge will be in coping with the growing needs while at the same time making sure that the investments to be made are compatible with the less than 2°C target. Consequently, this is where the bulk of the new infrastructure will need to be built to address the needs of the growing population, which in turn will require massive new investments in buildings and in energy, water and transport infrastructure. The first big push, or perhaps opportunity, will be in the massive investments that are projected to take place globally between 2015 and 2030. This growth is projected to be some US$90 trillion, which is higher than the current value of the existing infrastructure, which is estimated to be some US$50 trillion [47]. This infrastructure will have a long life, expanding some 50–100 years with major consequences for the carbon footprint for decades. The types of investments to be made on buildings, transport and mobility in general will also have an impact on the urban form of cities, which in turn will have a lasting influence of decades on the patterns of energy use and lifestyles of cities [48,49].

Drastically reducing the level of GHG emissions through investments in low-carbon infrastructure—the physical networks that are responsible for providing water, energy, transport, buildings, industry and waste management in cities—is, therefore, a prerequisite for the Paris Agreement targets. With a growing urban population, as already mentioned, large investments in infrastructure will be required to satisfy their demands, particularly in developing countries where the greatest growth will take place. It is here where two-thirds of the new investments will be needed and another third will need to be dedicated to replacing ageing infrastructure in developed countries [45]. What types of investments are made in urban infrastructure will also have, as already mentioned, an impact on the urban form or physical structure of cities and affect the systemic characteristics of urban energy use. This is turn has consequences and a lasting influence on the patterns of energy use in cities for decades to come and potential carbon lock-in [48]. A higher density urban form that promotes electric and non-motorized mobility and energy-efficient buildings will lead to lower energy use, in contrast to urban sprawl, which does not [50,51]. Path dependencies, particularly in transport, create challenges for policy-makers trying to introduce low-carbon goals. Vested interests often driven by political goals and economic growth priorities get in the way of policy-makers trying to break carbon lock-in through less motorized mobility [52].

In the sections above, the figure given for the investments required only for infrastructure to attend to the needs of the growing urban population is daunting. Much of the infrastructure to be built will have life spans of 50–100 years, thus dictating the carbon footprint for decades to come. Efforts to come up with criteria, metrics and principles for ensuring that these investments are compatible with the Paris Agreement are increasing, but more research and work on this is needed. The task is urgent given that, currently, the investment flows, particularly for infrastructure, are not nearly aligned with the targets of the Paris Agreement [43]. There are several approaches now being examined. One examines the possibility of coming up with 2°C investment criteria [53] and another uses sectoral and cross-sectoral emissions intensity thresholds to determine the compatibility of investment decisions with climate targets [54]. Another approach is that which is being taken by development finance institutions (DFIs). Their objective has been to integrate climate-related criteria in their analyses of project portfolios and their policies in general. These efforts have been around for some time and long before the Paris Agreement. The methodology and the discipline introduced for this analysis is nevertheless a good basis on which to make decisions more rigid and strict [55].

Several studies have examined the various technologies now available for decarbonizing the building sector. Recent studies also provide the windows of opportunity during which this decarbonization needs to take place. According to a recent study [56], in order for the 1.5° target to stay within reach, a fully decarbonized building stock by 2050 would be required. The technology now exists that provides savings in energy as high as 70–90%, and scenarios using state-of-the-art technologies and approaches show that energy savings of the order of some 46% by 2050 are possible; what is more important is that this can be done without major sacrifices to the quality of life of those residing in them [51]. In Latin America, particularly, the decarbonization of industry is also an important priority and is possible through efforts for greater efficiency and a greater use of renewable energy [57].

## 4. The implementation challenges: governance and institutions, climate finance and investments, and knowledge gaps

Given the complexity, magnitude and the scale of the climate change threat, and the importance of addressing the climate and development linkages, more integrated action and cooperation across sectors and geographies for more effective policy-making is essential. The following sections give a brief survey of some of the challenges that policy-makers encounter in pursuing these integrated approaches and cooperation. The number of challenges is large and varied. They range from issues of governance and institutions; to the lack of finance and the way in which climate finance becomes available; to the lack of technologies and know-how that are often not available at the right time and the right place; and lastly, to the lack of scientific data and information in certain areas, including knowledge on the linkages and interactions between climate and development and interlinkages between and among SDGs, as alluded to and briefly reviewed in the climate and development linkages section above.

### (a) Governance

Good and effective governance and strong institutional arrangements are key to the success of the Paris Agreement and the 2030 Agenda for Sustainable Development. The success and effectiveness of policy-making depend on the capacity of policy-makers to develop and implement programmes of action to address climate change and development in an integrated, coordinated and comprehensive manner and across sectors, geographies and constituencies. This applies to climate policy-making in general and to action in most sectors. The energy sector is one good illustration. Energy systems today are relying more and more on distributed generation systems. In these systems, a broad range of technologies and fuels, originating from various sectors, geographies and constituencies, interact to provide energy services. To increase efficiency and cost optimality, an overall energy systems perspective and integration are required [58]. Climate and development policy formulation in cities, where concerted action is so essential for the success of the Paris Agreement, is another good illustration of the governance challenges. Here the constraints of many cities are related to the multi-level governance which forces local policy-makers to depend on decision-makers at higher political levels and other actors [59–62]. Governance with devolution at the sub-national level, with policy-makers able to formulate policy and a regulatory framework, and who have control of the finances that would allow them to identify, formulate and implement projects across sectors, would seem essential [47,63]. Many cities unfortunately lack the political authority, the expertise, the finances and the institutional capacity to identify the best opportunities for low-carbon options and consequently for climate-related and development policies [64,65]. In all these cases, policy dialogue and interaction across various sectors, geographies and constituencies is what is required. But this is the challenge.

In this large, complex and fragmented landscape, understanding the political economy context of the location, region or country where policies, measures and regulations are to take effect is essential. Governance and institutions are established for managing with information, data, objectivity and fairness. But political and economic processes vary, often quite considerably, from locality to locality. The relationships and incentives are not always transparent or fair. Learning more about these dynamics is particularly important for climate change policies given the comprehensive set of factors that are involved, and that touch on so many communities and vested interests. The bottom line is that technical solutions are not enough. Often, the technical solution is obvious and straight forward. Implementing it is often a lot more complex. These factors need to be understood and taken into consideration. Fortunately, there is a growing body of literature that is providing methodology and guidance for this [66–68]. Can the Paris Agreement and the SDGs usher in a new era of institutional reform transformation? And what are the main features and characteristics of this new model of institutional system required for a major transformation to succeed? This is an area of research that is badly needed. From a preliminary survey of the literature, it appears that much literature exists on the general subject of climate governance and climate finance at the international level. The literature on climate and climate finance governance at the local level is much less abundant [69,70].

A review of the governance literature points to a fragmented institutional landscape that makes coordinated action difficult and sometimes even impossible [71–75]. A review of the literature also shows the various efforts to counteract this fragmentation to enable policy-makers achieve better climate policy integration and climate ‘mainstreaming’ into development. The field of climate policy integration (CPI) was born out of this concern and its growing literature is one that has begun to influence the debate of climate policy both in developed (in the EU particularly) as well as in developing countries [76–78]. Building on the more established field of environmental policy integration [79], the field of climate policy integration is defined as the effort to introduce climate policy objectives into other policy sectors while trying to minimize the potential contradictions in these policies [77]. In its Fifth Assessment Report, the IPCC reinforces the need for climate policy integration by calling for a type of development that combines both adaptation and mitigation as a way to promote the building of resilience [80]. Since this publication, more literature on climate policy integration has focused on the interactions between climate change mitigation and adaptation with application to the land use sector [81] and other policy domains such as forestry, agriculture, climate and energy [82].

### (b) Climate finance and investments

As the Fifth Assessment Report of the IPCC points out [64], there is no agreed definition of climate finance. In Chapter 16 on Cross-cutting Investment and Finance Issues, the IPCC adopted a broad definition of climate finance to include ‘all financial flows whose expected effect is to reduce net greenhouse emissions or to enhance resilience to the impacts of climate variability and the projected climate change.’ This broad definition includes domestic as well as international private and public funds, expenditure on mitigation and adaptation, and the costs of adaptation to climate variability and to future climate change. For the purposes here, this broader definition is the one used as it provides a better context for illustrating the significant increase and proliferation of funds and sources and the diversity of delivery systems at the national level. Given the magnitude of the investments needed, and the opportunities that these investments may bring, it is not surprising to see the growing number of actors, sources, funds, agents, investors, intermediaries, and channels growing at an impressive rate. While this is partly the result of a much-welcomed increase in the availability of climate finance, the challenge and the costs at the country level to manage this proliferation for the benefit of national climate change efforts is immense.

The Climate Policy Initiative (CPI) is one of the most comprehensive and scrutinized inventories of climate finance. Its latest report [83] gives an excellent overview of what sources and financial instruments are driving investments and how much climate finance is flowing globally. Understanding the how, where and from whom finance is flowing towards climate action and with what motivation, and how public and private sector sources of finance interact, is essential for the analysis of climate finance governance at the national and local levels. According to the most recent CPI report, some 62% of climate finance came from private sources ranging from households to multi-national corporations and their intermediaries. The total figure for this category was some US$243 billion, provided mostly by renewable energy investments. The actors in this category were diverse: some US$92 billion came from private project developers, the largest group by far; some US$58 billion was from corporate actors; US$43 billion came from households, a growing category; US$46 billion was provided by commercial financial institutions, private equity and venture capital; infrastructure funds provided some US$1.7 billion; and US$0.9 billion came from institutional investors.

Public sources including governments, multi-lateral and bilateral aid agencies and national DFIs were critically important however in this global picture because of their role in driving the global climate finance system through their knowledge and capacity-building activities and their role in reducing costs and risks. Some US$148 billion came from public sources, namely DFIs—national, bilateral and multi-lateral. Of this category, some US$66 billion came from national DFIs, the largest supplier of public climate finance at the national level; some US$47 billion came from multi-lateral DFIs; some US$17 billion from bilateral DFIs; US$15 billion from a variety of government agencies providing direct public contributions to climate projects; US$2 billion from climate funds; and US$1 billion from various risk management activities.

Despite their comprehensiveness, these figures do not capture much of the domestic resources dedicated to climate action, including those for adaptation and resilience (which is more directly linked to the broader SDG agenda and the climate and development linkage) or for investments in energy efficiency and other mitigation activities which are quite sizable and important. The figures provided focus mostly on renewable and clean energy investments with some general estimates of investments in other areas. It is nevertheless a useful breakdown of how much climate finance was invested, the orders of magnitude, the multiplicity of actors involved, the proliferation of funding sources, the instruments used, and the complementary roles of the public and private sectors. It is a very complex landscape indeed [64,84].

The proliferation of funding sources and the fragmentation of climate finance governance is one of the emerging issues that is posing serious challenges for policy coherence and effectiveness [71,72,74]. The current fragmentation and proliferation of climate finance makes it difficult and often impossible for policy-makers to formulate policies and programmes of action that cut across sectoral and constituency interests. Consequently, the much-needed joint approaches and integrated solutions for climate change are not always possible, and what is eventually offered and/or formulated is less than optimal. Many countries are experimenting with new institutional mechanisms to cope with the fragmentation and complexity of finance and governance. In the area of climate finance, for example, some countries are establishing national funding entities and national climate funds [85]. Others, such as Ethiopia, are among the very few that are addressing the fragmentation of governance of climate and development by merging these two agendas. Its Climate Resilient Green Economy (CRGE) vision and strategy adopted in 2011 is based on four pillars: improving agricultural practices while reducing emissions; building and regenerating forests with a focus on improving ecosystems services and carbon stock; increasing the share of renewable energy in final energy use; and introducing new technologies in transport, industry and building for better energy efficiency. The strategy promotes an economic development that pursues a low-emissions path while building resilience to adapt to climate change [86,87]. The government instruments for economic and social development, the Growth and Transformation Plans (GTP) I and II, aim for a high growth level but with climate neutral investments and policies [88]. In its NDC, Ethiopia reinforces its commitment by making extremely ambitious commitments to curb its GHG emissions by 64% from the business-as-usual scenario in 2030, focusing on a few sectors such as energy, buildings, water, agriculture, forestry and transport [89].

## 4. Conclusion

The extraordinary global achievements of the SDGs and the Paris Agreement provide the best opportunity yet for strengthening the linkages between climate and development. The evidence is quite clear. It is difficult if not impossible to reach the ambitious targets of the Paris Agreement without attending to the development implications. And it would be difficult if not impossible to reach the SDG goals and targets with worsening impacts of climate change. New approaches and methodologies are making the task of assessing these linkages more accessible. This includes the work on a new family of scenarios that tries to bring together analysis of biophysical as well as economic and social changes and incorporates qualitative analysis. The methodology being developed for assessing the linkages between and among SDGs will also make it possible for policy-makers to determine which policies are more suitable to their conditions.

The commitment of countries through their NDCs to act is a good first step but not nearly enough. These will need to be ramped up in terms of ambition based on the periodic assessment and review. The scientific evidence is quite clear as to the scale and magnitude of the action that needs to be taken and the small window of opportunity. One approach and an important step is to focus on those areas with a big impact and pay back in terms of emissions reductions. Some of these were shown here, for example the big projected growth in investments in urban infrastructure. Using the opportunity of the immense urban infrastructure projected for the next couple of decades, particularly in Africa and Asia, to ‘do it right’ from the start with low-carbon technologies would be one important way to make a major impact in decarbonization.

And, finally, issues of governance, institutions and fragmented finance will continue to pose major challenges for policy- and decision-makers. The hope is that the new demands brought about by bigger threats if action is not taken to address climate change and development effectively will force a new era of institution building and governance reform, as the Rio Conference did in the 1990s. In this respect, the emerging literature and debate on climate policy integration is encouraging.

## Data accessibility

This article has no additional data.

## Glossary

Climate finance: The AR5 of the IPCC WG III report adopts a broad definition of climate change that includes all financial flows whose expected effect is to reduce net GHG emissions or to enhance resilience to the impacts of climate variability and the projected climate change
Carbon footprint: The amount of GHG emissions produced to support human activities, either directly or indirectly
Carbon lock-in: The term refers to the pattern of carbon-intensive technologies to persist over time due to economic, technical and institutional factors, creating obstacles for lower carbon alternatives
Climate policy Integration: The field of climate policy integration is defined as the effort to introduce climate policy objectives into other policy sectors while trying to minimize the potential contradictions in these policies
Decarbonization: Reducing the carbon intensity. For example, the decarbonization of the power sector means reducing the emissions per unit of electricity generated
Low carbon urban Infrastructure: Infrastructure that is specifically designed to use significantly reduced amounts of energy and that emits either zero close to zero GHG emissions
Nationally determined contributions (NDCs): According to Article 4, paragraph 2, of the Paris Agreement [2], each Party is asked to prepare, communicate and maintain successive NDCs that it intends to achieve. Parties are also asked to pursue domestic mitigation measures, with the aim of achieving the objectives of such contributions. There will be a global stocktake every 5 years to assess progress with the first one to take place in 2018
Paris Agreement: The Paris Agreement entered into force on 4 November 2016. For the first time, and building on the UN Framework Convention on Climate Change, the agreement brings all the countries of the world together to combat climate change. The hope is that all nations will join without exception
Political economy: The analysis of political economy tries to understand the drivers of political behaviour which may include vested interests, power, incentives, informal social, political and cultural norms as well as values and ideas and the influence that this has on the formulation of policies
Sustainable development goals (SDGs): Also known as the 2030 Agenda for Sustainable Development, the SDGs were globally endorsed in 2015 and are composed of 17 broad development goals and 169 targets to be achieved by 2030
Urban form: This refers to the shape, size, density and configuration of built-up areas and transportation networks in a city
